# Various Uses of PD1/PD-L1 Inhibitor in Oncology: Opportunities and Challenges

**DOI:** 10.3389/fonc.2021.771335

**Published:** 2021-11-17

**Authors:** Zhitao Li, Guoqiang Sun, Guangshun Sun, Ye Cheng, Liangliang Wu, Qian Wang, Chengyu Lv, Yichan Zhou, Yongxiang Xia, Weiwei Tang

**Affiliations:** ^1^ Department of General Surgery, Nanjing First Hospital, Nanjing Medical University, Nanjing, China; ^2^ Research Unit Analytical Pathology, Helmholtz Zentrum München, German Research Center for Environmental Health (GmbH), Neuherberg, Germany; ^3^ Department of Geriatrics, The First Affiliated Hospital of Nanjing Medical University, Nanjing, China; ^4^ Hepatobiliary Center, The First Affiliated Hospital of Nanjing Medical University, Key Laboratory of Liver Transplantation, Chinese Academy of Medical Sciences, NHC Key Laboratory of Living Donor Liver Transplantation, Nanjing Medical University, Nanjing, China

**Keywords:** cancer, PD-1/PD-L1 inhibitors, drug combination therapy, immunotherapy, tumor microenvironment

## Abstract

The occurrence and development of cancer are closely related to the immune escape of tumor cells and immune tolerance. Unlike previous surgical, chemotherapy, radiotherapy and targeted therapy, tumor immunotherapy is a therapeutic strategy that uses various means to stimulate and enhance the immune function of the body, and ultimately achieves the goal of controlling tumor cells.With the in-depth understanding of tumor immune escape mechanism and tumor microenvironment, and the in-depth study of tumor immunotherapy, immune checkpoint inhibitors represented by Programmed Death 1/Programmed cell Death-Ligand 1(PD-1/PD-L1) inhibitors are becoming increasingly significant in cancer medication treatment. employ a variety of ways to avoid detection by the immune system, a single strategy is not more effective in overcoming tumor immune evasion and metastasis. Combining different immune agents or other drugs can effectively address situations where immunotherapy is not efficacious, thereby increasing the chances of success and alternative access to alternative immunotherapy. Immune combination therapies for cancer have become a hot topic in cancer treatment today. In this paper, several combination therapeutic modalities of PD1/PD-L1 inhibitors are systematically reviewed. Finally, an analysis and outlook are provided in the context of the recent advances in combination therapy with PD1/PD-L1 inhibitors and the pressing issues in this field.

## Introduction

Cancer mortality has remained high, owing to a lack of suitable cancer biomarkers for early detection. Many people are diagnosed at advanced stages, thus reducing the probability of successful treatment. The body’s immune system is critical in the battle against tumor. the process of enhancing the body’s immune system in order to limit tumor cell development or destroy tumor cells by controlling immune system activation through drugs or *in vitro* gene editing techniques. It is the fourth major field of anti-tumor therapy after surgery, chemotherapy, and radiotherapy, with the advantages of less adverse therapeutic effects, less damage to patient organs, more effective removal of residual tumor cells, and less likely to produce drug resistance (1). In 2013, immunotherapy of tumors was listed in the top ten scientific breakthroughs by Science magazine (2). Although several approaches to tumor immunotherapy have been developed, their effectiveness remains unsatisfactory due to numerous resistance mechanisms during tumor development, including systemic malfunction of T-lymphocyte-associated signaling, immunological tolerance building and local immunosuppression (3–5). It has been gradually realized that it is not enough to depress the “throttle” of the immune system, but it is also necessary to release its “brake”, which has led to the creation of Immune checkpoint blockers. Among them, PD-1/PD-L1 inhibitors has performed particularly well and has changed the treatment landscape of many advanced tumors. While the use of PD1/PD-L1 inhibitors alone can lead to long-term remission and improve survival prognosis in some populations, a significant number of patients fail to generate an effective response. To increase tumor response rates and improve prognosis, immune combination therapy may be one of the effective strategies (6). The combination of immunotherapeutic drugs and other drugs may raise the tumor’s immunogenicity and thus enhance the effect of them (7–9).

## Regulation of PD1/PD-L1 Expression and Drug Resistance Mechanisms

PD-1 is a transmembrane receptor mainly expressed on activated T cells, B cells, mesenchymal stem cells, natural killer cells, and monocytes (10). Its ligand, PD-L1, is substantially more abundant in tumor tissues than in normal tissues and is found on the surface of the majority of tumor cells (11–13). PD1/PD-L1 is an important immune check signaling pathway, which can be used by tumor cells to block T cell activation in order to avoid immunological onslaught (14). Inhibitors that block the PD-1/PD-L1 signaling pathway can activate anti-tumor immunity, and effectively inhibit tumor growth or even cure tumors (15). But the effectiveness of PD1/PD-L1 inhibitors depends on their expression on the cell surface. For tumors with low or no expression of the corresponding substances, the inhibitors do not work well. Their expression is influenced by transcriptional, translational, post-transcriptional and post-translational factors, and PD1/PD-L1 expression is regulated by several signaling pathways, thereby affecting the immune response of tumor cells and the efficacy of clinical anti-PD-1/PD-L1 therapy. Understanding the mechanisms of resistance, and screening patients for potential benefit are new directions in research to increase the curative effect of PD1/PD-L1 inhibitors.

### PD-1 Regulatory Mechanisms

During the transcription and expression of Pdcd1, the gene encoding PD-1, two conserved regions (CR-B and CR-C) located upstream of the transcription start site (TSS) of the Pdcd1 gene play an important role (16). Researchers found that there are regulators in both transcriptional regulation and epigenetic regulation of PD-1 expression that are closely associated with these two conserved regions. In terms of transcriptional regulation, the CR-B region contains binding sites for activator protein 1 (AP-1) and the negatively regulated transcription factor T-bet.AP-1 is a class of four sub AP-1 is a class of transcriptional activators consisting of four subfamilies (Fos, Jun, ATF and Maf) (17). It is involved in various cellular processes, including cell differentiation, proliferation and apoptosis, by regulating the expression of related target genes (18). Xiao et al. (19) found a large amount of AP-1 subunit protein c-Fos in complex with JunB on tumor-infiltrating T cells, and found that the latter bound to the AP-1 binding site in the CR-B region, resulting in upregulation of PD-1 expression. T-bet was the first regulatory factor identified to have the ability to repress Pdcd1 transcription. Further studies have demonstrated that it can negatively regulate PD-1 transcription by binding directly to the corresponding site in the CR-B region after stimulation by TCR signaling (20). However, the researchers found that PD-1 expression in CD8+ T cells was only slightly increased in T-bet knockout mice after viral infection compared to the comparison group, which also suggests that T-bet may need to synergize with other factors to co-inhibit PD-1 expression. Compared with the CR-B region, the CR-C region has a richer set of regulatory sites, including nuclear factor of activated T-cells cytoplasmic 1 (NFATc1) binding sites, IFN-stimulated response element (ISRE), nuclear factor-κB (NF-κB) binding site and forkhead transcription factor 1 (FoxO1) binding site (21). The persistence and strength of the TCR signaling pathway affects the expression of PD-1 in CD8+ T cells, where the TCR/Calcineurin/NFATc1 pathway is considered to be one of the major initiating upstream signaling pathways that activate PD-1 transcriptional expression. The transcription factor NFATc1 binds to its counterpart element within the CR-C region upon stimulation by TCR signaling to initiate PD-1 transcription, which in turn promotes PD-1 expression (16). ISRE is a PD-1 regulatory element located within the CR-C region, and its activation is closely associated with the generation of the IFN-stimulated gene factor 3 (ISGF3) complex. When IFN-α activates the JAK/STATs signaling pathway, interferon response factor 9 (IRF9) and STAT1-STAT2 heterodimers combine to form ISGF3 and then bind to ISRE to promote PD-1 expression on CD8+ T cells and macrophages (22). And for CD8+ T cells alone, Terawaki et al. (23) found that treatment of T cells with IFN-α alone did not promote PD-1 expression, but when activated *via* the TCR signaling pathway, treatment of T cells with IFN-α enhanced PD-1 expression. The TCR/Calcineurin/NFATc1 signaling pathway was able to induce T cell transcription of Pdcd1 in macrophages, but not in macrophages. Bally et al. (24) found that during activation of macrophages, Pdcd1 transcription was activated by NF-κB, and the p65 subunit of NF-κB was able to bind to the corresponding element within the CR-C region and upregulate PD-1 expression. In the early stages of chronic infection, NFATc1 is a regulator that activates Pdcd1 transcription in CD8+ T cells. Agnellini et al. (25) found that NFATc1 is unable to enter the nucleus of CD8+ T cells after chronic infection is established, when Pdcd1 transcription is activated by FoxO1. DNA methylation of the gene promoter region represses gene transcription (26). In terms of epigenetic regulation, CR-B and CR-C were fully methylated in naive T cells that did not express PD-1, whereas during the differentiation of naive T cells to effector CD8+ T cells after viral infection, PD-1 expression levels were continuously upregulated and the methylation levels of these two regions gradually decreased, with a significant negative correlation between them (27). This also suggests that changes in methylation in the CR-B and CR-C regions have an important regulatory role on PD-1 expression.

Outside of these two major regulatory regions there are also a number of role elements involved in the regulation of PD-1 expression, such as those located at the B lymphocyte induced maturation protein-1 (B lymphocyte-1, Blimp-1) binding site, signal (B lymphocyte induced maturation protein-1, Blimp-1) binding sites, signal transducers and activators of transcription (STATs) binding sites, recombination signal binding protein-Jk (RBPJk) binding sites associated with the Notch signaling pathway, and Blimp-1 is a transcriptional repressor that functions similarly to T-bet, and when activated by T cells, Blimp-1 binds to the binding site between CR-C and CR-B and directly affects the inhibition of Pdcd1 transcription. In addition to this, Blimp-1 can also inhibit the expression of NFATc1 (the transcriptional activator of Pdcd1). And through this feed-forward loop can rapidly shut down the transcription of Pdcd1 thereby inhibiting the expression of PD-1 (28). STAT3 (or STAT4) is able to bind to the 5′ and 3′ distal sites of the TTS of the Pdcd1 gene and directly regulate the transcriptional expression of PD-1 in activated CD8+ T cells (29). In addition, Mathieu et al. (30) found the presence of RBPJk binding sites upstream of CR-C, which upon binding were able to activate the transcriptional expression of Pdcd1. Two other important Pdcd1 regulatory elements are the isolators (insulators) located on either side of Pdcd1 (-25.7 kb and +17.5 kb). They are two terminals of Pdcd1 cis-regulation,which bind the corresponding transcription factor (CTCF) upon NFAT stimulation respectively, thereby altering the topology of the regulated gene and preventing activation of unrelated genes by enhancers (29) (**Table 1**).

**Table 1:** Regulation mechanism of PD-1.

Type of regulation	Regulator	Key process	Regulation effect	Reference
**Transcriptional regulation**	TCR/Calcineurin/NFATc1 signaling pathway	NFATc1 activation	Positive	(16)
	STAT3(IL-6) and STAT4(IL-12)、AP-1	Binding to the corresponding part of the PDCD1 gene	Positive	(19, 29)
	JAK/STATs signaling pathway	Formation of IFN-stimulated gene factor 3 (ISGF3)	Positive	(23)
	Notch signaling pathway	Formation of RBPJk and Notch1 intracellular domain (NICD)	Positive	(22)
	PI3K/AKT/mTOR signaling pathway	The decreased signaling of the pathway increased FoxO1 activity	Positive	(31)
	T-bet、Blimp-1	Binding to the corresponding part of the PDCD1 gene	Negative	(20, 32)
	NF-κB signaling pathway	The expression of NF-κB increased	Positive	(33)
**Post-transcriptional regulation**	miR-138、miR-28	Acting on the 3'-UTR of PD1 mRNA	Negative	(22, 34)
**Post-translational modification**	FBXO38	Promoting ubiquitination of PD-1 protein	Negative	(35)
**Epigenetic regulation**	Special AT-rich sequence binding protein (SATB-1)	Recruiting a nucleosome remodeling deacetylase (NuRD) complex	Negative	(36)
	conserved regions (CR-B and CR-C)in Pdcd1	Lower level of methylation	Positive	(27)

### PD-L1 Regulatory Mechanisms

PD-L1 expression in tumor cells is not only regulated by pro-oncogenic signaling pathways, but also influenced by multiple factors in the tumor microenvironment (TME) and multiple intracellular enzymes (37). At present, the mechanisms regarding the regulation of PD-L1 in cancer can be grouped into four categories, namely regulation through genomic alterations and rearrangements, signaling pathways, miRNAs and post-translational modifications.

Intra-tumor gene mutation and amplification are associated with the regulation of PD-L1. When located on chromosome 9p24.1 After amplification and translocation of CD274, the gene encoding PD-L1, in some tumors (primary mediastinal large B lymphocytoma, non-small cell lung cancer, squamous cell carcinoma and EBV-positive gastric cancer) there may be an increase in PD-L1 expression levels (38, 39).

**Figure 1:**
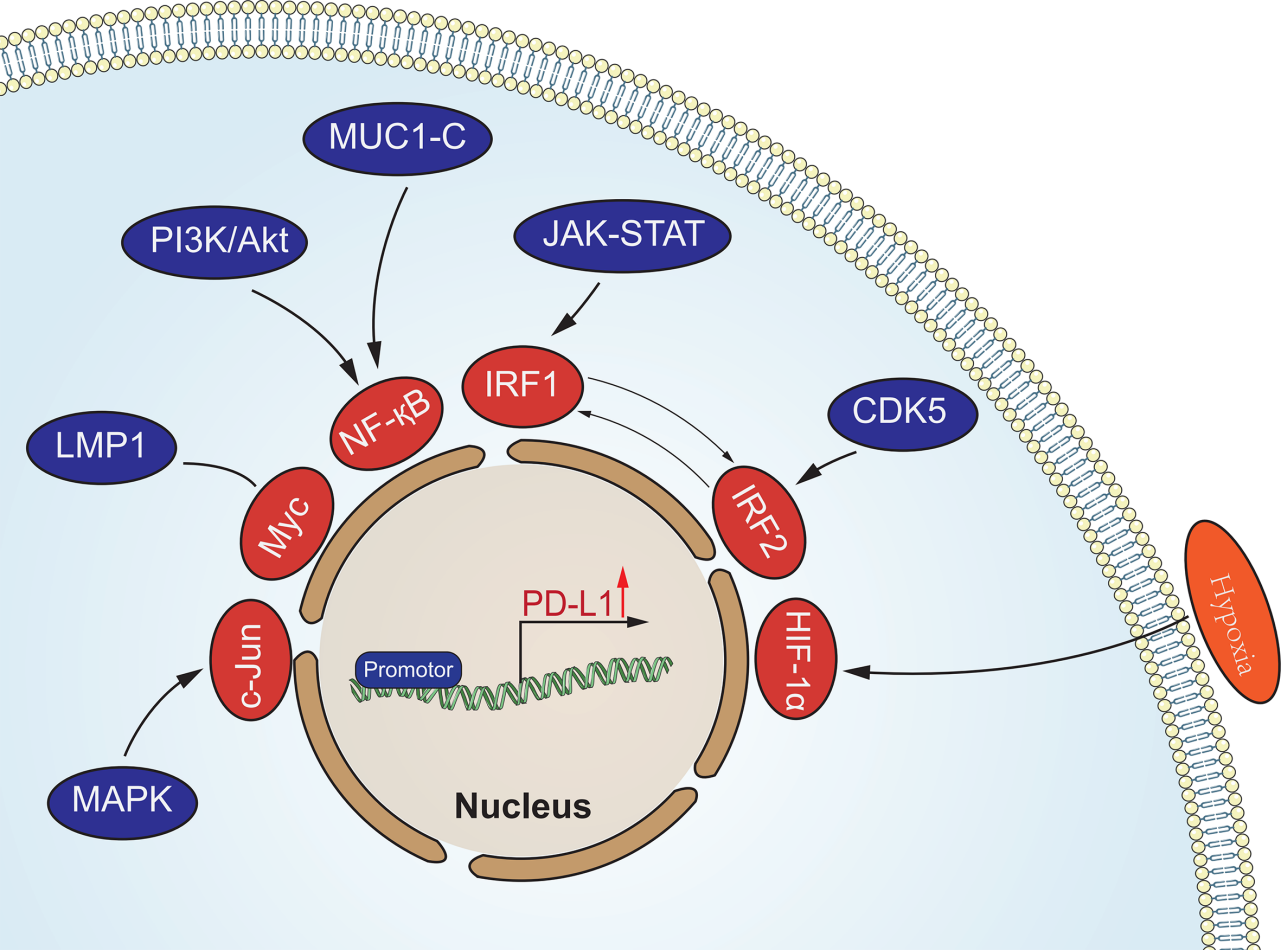
Oncogenic signaling pathways in tumors involved in the regulation of PD-L1 expression. These signaling pathways regulate the expression of PD-L1 at the transcriptional level.

Oncogenic signaling pathways in tumors are not only involved in the regulation of tumorigenesis and progression, but also in the regulation of PD-L1 expression to assist tumor cells in generating immune escape (**Figure 1**). This regulation is mainly involved through signaling transcriptional regulators, pathway effector molecules and upstream receptors. Many transcriptional regulators are involved in the direct transcriptional regulation of PD-L1, for example, Myc can promote PD-L1 expression by binding to the promoter of the PD-L1 gene (40). It has also been reported that MYC binding to the PD-L1 promoter leads to downregulation of PD-L1 expression, which enhances tumor immunocidal capacity (41). Nuclear transcription factor-κB (NF-κB) is a transcription factor widely expressed in various cell types, which can be activated by multiple inflammatory signaling pathways and oncogenic mutations. Tumor necrosis factorα (TNF-α) is a type II transmembrane protein that has a strong killing effect on tumor cells and is also one of the important activators of NF-κB. Asgarova (42) found that when lung cancer cells undergo epithelial- mesenchymal transition (EMT) during treatment with TNF-α resulted in NF-κB recruitment at the promoter of PD-L1 and upregulation of PD-L1 expression. It was shown that RelA, a subunit of NF-κB, is able to bind to the PD-L1 promoter region, further suggesting that NF-κB can be directly involved in the regulation of PD-L1 expression (43). STAT3 is a transcription factor that plays a key role in the production of immunosuppressive factors and promotion of cancer cell proliferation in the tumor microenvironment. Atsaves (44) STAT3 is a transcription factor that plays a key role in the production of immunosuppressive factors and promotion of cancer cell proliferation in the tumor microenvironment. Hypoxia is a common feature of most solid tumors, and hypoxia activates the expression of downstream genes by promoting the expression of hypoxia-inducible factors (HIF-1α and HIF-2α). HIFs can directly bind to and induce transcription of the hypoxia response element (HRE) of the PD-L1 promoter region (45). Thus overexpression of HIF-1α leads to upregulation of PD-L1 expression. In addition to binding to the promoter region, AP-1 is able to bind the enhancement region of the first intron of PD-L1 to regulate the transcription of PD-L1 (46). Cyclin-dependent kinase 5 (CDK5) is a non-classical cyclin protein kinase capable of promoting several processes such as angiogenesis, apoptosis, and vesicle transport.IRF2 and IRF2BP2 are PD-L1 transcriptional repressors and CDK5 can regulate PD-L1 transcription by repressing the expression of both and thereby regulating PD -L1 transcription (47). In addition, activation of MAPK signaling pathway and PI3K signaling pathway promotes PD-L1 expression by activating transcription factors c-Jun and Akt respectively.

MicroRNAs (miRNAs) are a class of small non-coding single-stranded RNAs consisting of 20-22 nucleotides, which cause degradation or translation inhibition of target gene mRNA by targeting the untranslated region (3’-UTR) at the 3’ end of the mRNA (48). Several reports have shown that mi RNA can miRNA regulates PD-L1 expression in tumors by targeting the 3’-UTR of PD-L1 mRNA, including gastric cancer, pancreatic cancer, ovarian cancer, non-small cell lung cancer, and leukemia (49–52). Among these miRNAs, most of them repress PD-L1 expression by binding to PD-L1 mRNA, such as: miR-93, miR-142-5p, miR-138-5p, miR-106b, miR-200, miR-217, miR-570, miR-152, miR-15a, miR-17-5p, miR-193a and miR-16. And Zhu et al. (53) found that miR-130b, miR-20 and miR-21 indirectly promoted PD-L1 expression in colon cancer by inhibiting PTEN expression.

Post-translational modification (PTM) refers to post-translational chemical modifications of proteins, including phosphorylation, ubiquitination, acetylation, glycosylation and methylation. Precursor proteins are inactive and often undergo a series of post-translational processing to become functional mature proteins.PD-L1 is modified by different post-translational modifications that affect its localization, stability, and pathological and physiological functions (54). Li et al. (55) found that glycogen synthase kinase 3 beta (GSK3β) can directly bind to the C-terminal structural domain of PD-L1, catalyze the phosphorylation of the T180 and S184 sites of PD-L1 and then be recognized by Beta-transducin repeats-containing proteins (β-TRCP), and ultimately promote the polyubiquitination and degradation of PD-L1. Ubiquitination is the process by which ubiquitin molecules sort intracellular proteins by a series of specific enzymes, from which target protein molecules are selected and specifically modified. Zhang et al. (56) found that CDK4 can negatively regulate PD-L1 by mediating SPOP protein phosphorylation to indirectly ubiquitinate PD-L1 for degradation. While Chemokine-like factor MARVEL transmembrane domain-containing family (CMTM) family members CMTM6 and CMTM4 not only enhance PD-L1 protein half-life and inhibit PD-L1 ubiquitination, but also protect PD-L1 from lysosomal-mediated degradation. N-glycosylation is key protein modification that determines protein structure and function (especially membrane protein function). The extracellular region of PD-L1 (N219, N200, N192, N35) is a glycosylation binding site, and glycosylation prevents GSK3β-induced leading to PD-L1 phosphorylation and ubiquitination, which can play a role in stabilizing PD-L1 (57) (**Table 2**).

**Table 2:** Regulation mechanism of PD-L1.

Type of regulation	Regulator	Key process	Regulation effect	Reference
**Genetic variation**	PD-L1 genome	Amplification	Positive	(58)
	PD-L1 genome	Chromosome ectopia	Positive	(59)
**Transcriptional regulation**	NF-κB signaling pathway	The expression of NF-κB increased	Positive	(33)
	HIF-1α signaling pathway	The expression of HIF-1α increased	Positive	(60)
	IFNγ signaling pathway	Activating IRF1 and IRF2	Positive	(61, 62)
	PI3K/Akt signaling pathway	Loss of PTEN causes PI3K to activate	Positive	(63)
	MAPK signaling pathway	Activating c-Jun	Positive	(64–66)
	Hippo signaling pathway	Hippo pathway inactivation	Positive	(67)
	c-Myc	C-Myc overexpression	Positive	(68)
**Post-transcriptional regulation**	miR-513, miR-570, miR-34a, miR-873, miR-424 and miR-200	Acting on the 3'-UTR of PD-L1 mRNA	Negative	(50–52, 69, 70)
	miR-18a, miR-23a-3p, miR-200a	Inhibiting PTEN expression	Positive	(71–74)
**Post-translational modification**	Beta-transducin repeats-containing protein (β-TRCP)	Promoting ubiquitination of PD-L1 protein	Negative	(39)
	CKLF like MARVEL transmembrane domain containing 6 (CMTM6)	Inhibiting ubiquitination of PD-L1 protein	Positive	(75)
	COP9 signalosome complex subunit 5 (CSN5)	Promoting deubiquitination of PD-L1 protein	Positive	(55)
	GSK3β、Metformin	Promoting phosphorylation of PD-L1 protein	Negative	(76, 77)
	IL-6 / JAK1 pathway	Promoting phosphorylation of PD-L1 Y112	Positive	(78)
	BetaGalbeta-1,3-Nacetylglucosaminyltransferase 3 (B3GNT3)	Promoting glycosylation of PD-L1 protein	Positive	(77)
**Epigenetic regulation**	MLL1-H3K4me3 Axis	Histone methylation	Positive	(79)
	EZH2	Histone methylation	Negative	(80)
	Methylation of some CpG loci in CD274 promoter	DNA methylation	Negative	(81, 82)
	histone deacetylase (HDAC)	Histone acetylation	Positive	(83)

### PD-1/PD-L1 Resistance Mechanisms

**Figure 2:**
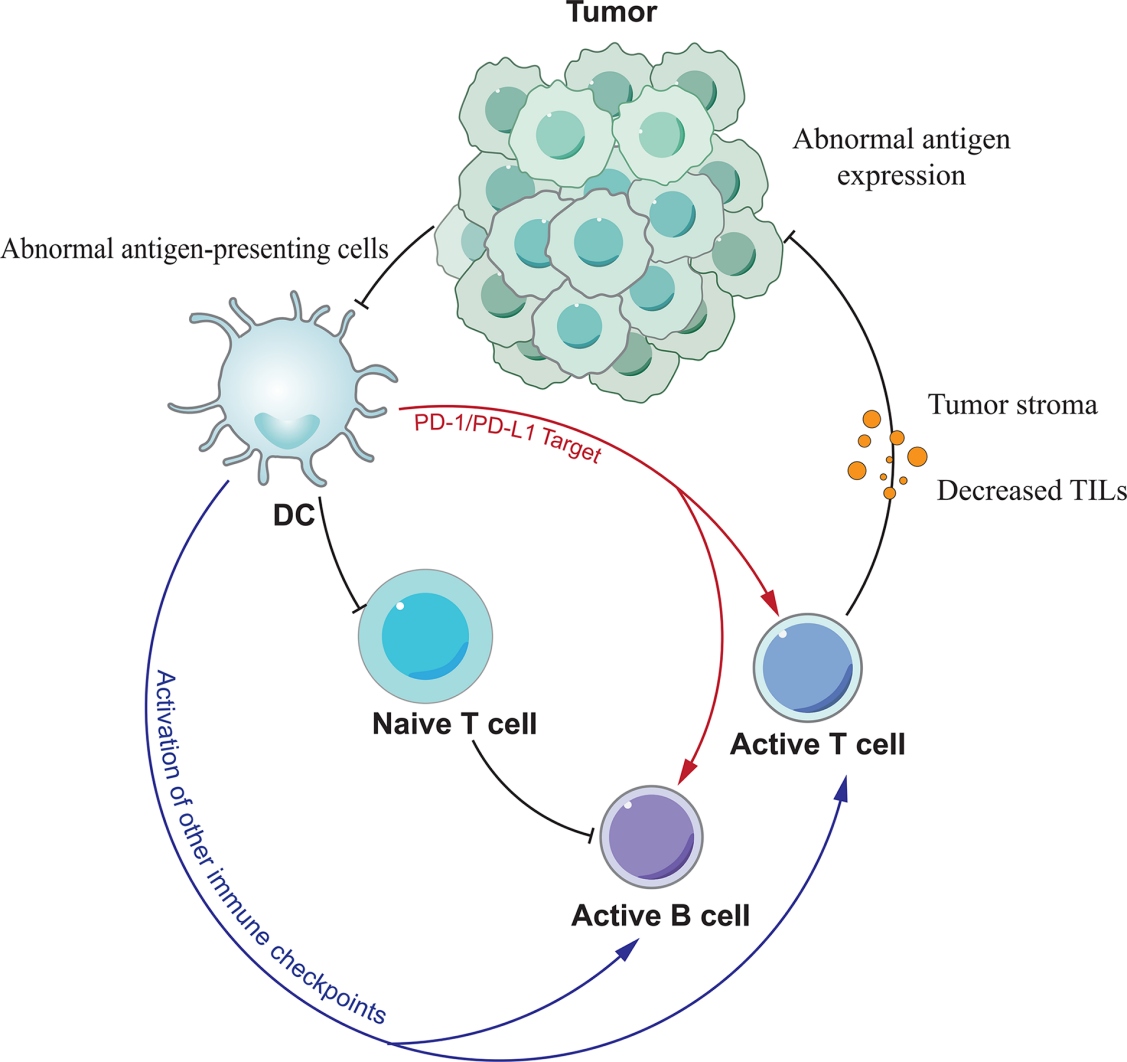
The mechanism of PD-1/PD-L1 antibody resistance. Various factors lead to resistance mainly by affecting the function of T cells.

Tumor immunotherapy represented by PD-1/PD-L1 monoclonal antibodies has opened a new era of tumor treatment. However, immunotherapy has a high rate of drug resistance compared with molecular targeted therapy and chemotherapy, which is a problem that cannot be ignored in clinical practice (84). The development of PD-1/PD-L1 antibody resistance is a complex, dynamic and interdependent process, with many intrinsic and extrinsic tumor cell factors associated with it (**Figure 2**). Resistance can be classified into secondary and primary resistance according to the time of its emergence. Secondary resistance refers to the emergence of resistance after the initial treatment has been effective in controlling tumor progression. Primary resistance, on the other hand, exists prior to exposure to the drug and prevents the drug from impeding tumor progression (85). PD-1/PD-L1 resistance has been shown to be associated with PD-1/PD-L1 expression levels on the cell surface, the absence of first and co-stimulatory signals, the tumor microenvironment, and epigenetic modifications.

Although PD-1 or PD-L1 inhibitors can block the linkage of both, they cannot affect the expression of PD-1/PD-L1 on the cell surface, and the high expression of PD-1 on the T cell surface may be related to PD-1/PD-L1 inhibitor resistance. In the tumor microenvironment tumor cells and antigen presenting cells (APCs) upregulate PD-L1 expression through 2 pathways, adaptive immune tolerance and intrinsic immune tolerance, to evade immune surveillance, and PD-L1 upregulation is also a prerequisite for the efficacy of anti-PD-1/PD-L1 monoclonal antibodies. Juneja et al. (86) have demonstrated through mouse experiments that PD-L1 expression on tumor cells and immune cells levels play a key role in the efficacy of PD-1 antibodies. Therefore, the expression of PD-1/PD-L1 can be influenced by modulating some of the regulators described above, thereby improving the response to PD-1/PD-L1 inhibitors in tumor or non-tumor patients.

Unlike normal cells, tumor cells possess immunogenicity because they mutate to induce the expression of specific antigens on the cell surface. Tumor antigens are acquired by antigen presenting cells (APCs) and presented to T cells. Activated T cells then play a therapeutic role. The most direct reason for the failure of PD-1/PD-L1 antibodies to treat tumors is the inability of T cells to recognize them due to the lack of tumor antigens. Sade-Feldman et al. (87) found that mutations or deletions of beta-2-microglobulin (B2M) resulted in the loss of MHC I expression and impaired antigen recognition, resulting in the inability of cells to recognize tumor cells, which in turn led to drug resistance. Tumors can reduce the number of APCs by secreting inhibitory factors such as vascular endothelial growth factor (VEGF) and interleukin-10 (IL-10). When APC is abnormal, it also leads to impaired signaling, which in turn leads to abnormal T-cell recognition. In addition to the first signal, T cells require a second signal, namely co-stimulatory signals, to exert their immune action. After evaluating the effects of CD40 agonists using a chronic infection model, Xu et al. (88) found that CD40 agonists could enhance the therapeutic effects of PD-1 inhibitors by transforming inactivated PD-1-expressing cytotoxic T lymphocytes (CTL) through activation of the mTORC1 pathway. This also suggests that co-stimulatory signaling stimulators can enhance T-cell function. And when co-stimulatory signaling is absent, the efficacy of PD-1/PD-L1 inhibitors is reduced and drug resistance develops.

The tumor immune microenvironment is dynamically regulated by the interaction of tumor tissue and associated immune cells, providing a chronically inflammatory and immunosuppressive environment for tumor survival and progression. In addition to tumor cells, components that may be associated with primary or acquired drug resistance include tumor-infiltrating lymphocytes, CD8+ T-cell depletion, tumor-associated immunosuppressive cells, and other suppressive immune checkpoints. Tumor infiltrating lymphocytes (TIL) are the effector cells of the immune response and the effect of antitumor immunity is related to their activity and number. Tumeh et al. (89) found that the presence of CD8+ T cells in the tumor was a prerequisite for tumor shrinkage after treatment with anti-PD-1/PD-L1 monoclonal antibodies in metastatic melanoma, and that chemokine expression played a key role in the migration of T cells from the circulatory system into the tumor. When the number of tumor-infiltrating lymphocytes is reduced the effect of immunotherapy can be diminished or even lead to drug resistance. In the tumor setting, CD8+ T cells show upregulation of PD-1 expression in response to sustained antigen stimulation, and PD-1 interacts with PD-L1 to form a depleted T cell phenotype. Anti-PD-1/PD-L1 monoclonal antibodies were able to reverse PD-1 moderately expressed failing CD8+ T cells, but were ineffective against severely failing CD8+ T cells with high PD-1 expression (90). This suggests that the key to reversing PD-1/PD-L1 antibody resistance may lie in the ratio between the two. Tumor-associated immunosuppressive cells located in the tumor stroma include bone marrow-derived suppressor cells (MDSCs), regulatory T cells (Tregs) and M2 macrophages. They diminish the effect of anti-PD-1/PD-L1 monoclonal antibodies by suppressing antitumor effector T cell function. MDSCs are major regulators of immune responses in various pathological conditions, and they can promote tumor invasion and metastasis by promoting angiogenesis. In addition, MDSCs present in the tumor microenvironment reduce the effectiveness of immunotherapy (91). Tregs suppress effector T cell function by secreting suppressive cytokines (TGF-β, IL-35, and IL-10). Ngiowet et al. (90) found that Tregs promote CD8+ T cell depletion and the CD8+/Tregs ratio can be used as a predictor of anti-PD-1 monoclonal antibody efficacy. And M2 macrophages are tumor-associated macrophages (TAMs) with pro-cancer properties. Clearance of these cells may enhance the effect of anti-PD-1/PD-L1 monoclonal antibodies. In addition to PD-1/PD-L1, other immune checkpoints such as TIM-3, CTLA-4, LAG-3 and IDO are also involved in regulating the immune response, and they also have an impact on PD-1/PD-L1 antibody efficacy (92). For example, tumor recurrence after PD-1/PD-L1 antibody treatment may be the result of increased TIM-3 expression in T cells. In contrast, LAG-3 monoclonal antibody can restore the body’s anti-tumor immune activity by reducing the number of bone marrow-derived suppressor cells (MDSC), inhibiting the activity of Treg cells in the tumor microenvironment and increasing the number of activated CD8+ T cells (93).

In terms of epigenetic modifications, the epigenetic factors histidine-lysine N-methyltransferase (enhancer of zeste homolog 2, EZH2) and DNA methylation may be important mechanisms mediating immunotherapy resistance. Emran et al. (94) found that DNA hypermethylation inhibits the endogenous interferon response required to recognize cancer cells, leading to immune suppression. While hypomethylation also leads to increased expression of the inhibitory cytokines VEGF, IL-6 and PD-L1 and thus affects the efficacy of immunotherapy. In contrast, DNA methyltransferase inhibitors can reverse immunosuppression by affecting antigen processing and delivery and enhancing the expression of other immune-related genes, co-stimulatory molecules, tumor-associated antigens and chemokines (95). In addition, DNA methylation has been found to cause T-cell depletion, and by reversing the depleted state of T cells can promote the restoration of T cells to their immune function (96).

## Combining With Other Immune Checkpoint Drugs

The introduction of immune checkpoint inhibitors (ICIs) as a “brake” on the immune system has provided a new option for patients with advanced cancer, and PD-1/PD-L1 inhibitors, the most commonly used immune checkpoint inhibitors, are widely used in the treatment of various cancers. However, as research has progressed, it has been found that it does not benefit all patients with advanced cancer, with only a fraction of patients showing a response. Even when PD-1 and ligands are present, many parallel immune checkpoint pathways cause anti-PD1/PD-L1 treatment tolerance (97). Combining other immune checkpoint pathway inhibitors can have the effect of increasing the sensitivity to drug therapy. Curran et al. (98) found that treatment with antibodies combining two immune checkpoint molecules improved the antitumor response in preclinical animal models. Immune checkpoint inhibitors that have been reported to be used in combination are CTLA-4 inhibitors, TIM-3 inhibitors, LAG-3 inhibitors, indoleamine 2,3 -dioxygenase (IDO) inhibitors, TIGIT inhibitors, B7-H3 monoclonal antibodies, and VISTA inhibitors.

### CTLA-4 Inhibitors

CTLA-4 is a transmembrane protein on the surface of T cells, expressed on the surface of activated CD8+ T cells, CD4+ T cells and Treg cells (99). And it is a key inhibitory receptor affecting T cell function. CTLA-4 inhibitors efficiently bind CTLA-4 molecules, reducing the number of CTLA-4 molecules bound to B7 molecules, and inhibiting T cell activation by reducing the production of negative regulatory signals, enhancing T cell activation and the growth and infiltration of tumor-differentiated T cells in the tumor microenvironment, thereby achieving antitumor effects of the immune system. Some CTLA-4 inhibitors have been approved by the Food and FDA for clinical use with promising results (100). And representative drugs include Ipilimumab and Tremelimumab. PD-1 acts in the late stage of T-cell activation, after T-cell migration into the tumor microenvironment, while CTLA-4 acts in the early stage of T-cell activation (101). Beavis et al. (102) found that CD4+Foxp3-T cells are effector T cells with anti-tumor functions, and increasing the number of CD4+Foxp3-T cells in tumor tissue can improve the effectiveness of tumor treatment. Both CD8+ T cells and CD4+Foxp3-T cells could be activated when CTLA-4 and PD-1 were blocked simultaneously. This provides a rationale for the use of CTLA-4 in combination with PD-1/PD-L1 inhibitors for cancer treatment. This combination approach has become the most intensively studied as well as the most widely used immune combination regimen (103). Daniel et al. (104) evaluated the efficacy of Pembrolizumab plus Ipilimumab in melanoma patients after failure of anti-PD-1/PD-L1 therapy and showed that 29% of patients achieved a confirmed response (including 21.4% partial responses and 7.2% complete responses). This demonstrates the significant antitumor activity of the combination. A study of long-term follow-up of patients with advanced melanoma treated with Nivolumab Plus Ipilimumab showed that these patients had a 3-year Overall Survival (OS) of 63% (105). This also demonstrates the effectiveness and durability of the combination of the two. Although combination therapy showed multiple advantages, in terms of safety, it had a higher incidence of treatment-related adverse events than chemotherapy (24.5% *vs*. 13.9%), and discontinuation due to treatment-related adverse events was more common (18.1% *vs*. 9.1%) (106). In a meta-analysis of solid tumors, the probability of immunotherapy-related adverse reactions following treatment with nivolumab in combination with ipilimumab was higher than with nivolumab alone. of these adverse reactions, the incidence of grade 3+ gastrointestinal toxicity (9.9% *vs*. 1.2%) and hepatotoxicity (10.1% *vs*. 1.3%) were significantly higher (107). In addition, a study of non-small cell lung cancer treatment showed that the incidence of grade 3 or higher treatment-related adverse events was 33.8%, 22.9%, and 14.9% in the chemotherapy group, the Durvalumab combined with Tremelimumab group, and the Durvalumab group, respectively (108). Different dosing sequences, drug doses, and dosing intervals in different tumors can affect the frequency and severity of adverse events. Sen et al. (109) demonstrated that the incidence of immune-related adverse events (irAEs) increased with increasing doses of immune checkpoint inhibitors, but that disease control rate (DSR), OS, and PFS did not improve. This also justifies the low-dose immune checkpoint inhibitor regimen. According to the results of the Checkmate012 study, low-dose combinations may hold promise for reducing the incidence of adverse events and improving clinical efficacy (103). In a retrospective analysis of 134 unselected stage IV solid cancer patients, Kleef et al. (110) found that the use of off-label low doses of nivolumab plus ipilimumab combination did not compromise efficacy and that the post-treatment emerged with a safer irAEs profile. Research on such combinations is currently in full swing, and in the future the optimal dosing order, dosing interval, and dosing review rate for different tumors may be answered.

### TIM-3 Inhibitors

T-cell immunoglobulin adhesion protein molecule 3, a member of the Tim family, is a type I transmembrane glycoprotein.TIM-3 is widely distributed on the surface of T cells, regulatory T cells, and innate immune cells (DC cells, NK cells, monocytes). It was first found to be expressed at high levels in “depleted” T cells of HIV-infected patients and was later shown to be an important player in T cell depletion (111). TIM-3 can lead to T cell depletion and contribute to tumor immune escape, invasion and metastasis, thus affecting patient outcome and prognosis. It is worth mentioning that although TIM-3 is considered to be a suppressor receptor, one study found that TIM-3 can also play a role in activating T cells (112). Sakuishi et al. (113) found that on most tumor infiltrating lymphocytes (TILs) in mice bearing solid tumors,and there was co-expression of PD-1 with TIM-3. They also found that the combined use of anti-PD-1 and anti-TIM-3 significantly slowed down tumor growth, demonstrating the effectiveness of combined use of PD-1 and TIM-3 inhibitors in controlling tumor growth. Currently, TIM-3 inhibitors such as TSR-022, MBG453, Sym023, LY3321367, INCAGN-02390, and BGB-A425 have entered clinical trials, and researchers need to conduct numerous trials before they can conclude whether their combination with PD-1/PD-L1 inhibitors in clinical use can meet expectations.

### LAG-3 Inhibitors

LAG-3, also known as CD223, is a type I transmembrane protein that is also a member of the immunoglobulin superfamily. It is mainly expressed on the surface of activated CD4+ T cells and CD8+ T cells, but also on the surface of other immune cells such as B cells, natural killer cells and dendritic cells (114). However, under physiological conditions, naive and normal T lymphocytes express only low levels of LAG-3. After tumor antigen stimulation, the level of LAG-3 expression in lymphocytes increases substantially (115). At the same time, its expression level was positively correlated with tumor development, suggesting that interruption of LAG-3 may enhance anti-tumor immunity.LAG-3 not only inhibits T cell activation and proliferation, but also promotes the activity of regulatory cells (Tregs). This allows LAG-3 antibodies to simultaneously restore T-cell function and suppress Treg cell activity. In contrast, according to previous studies antibodies against PD-1 can only restore T-cell function (116). This has led researchers to see the value of the research behind it. Woo et al. (117) found extensive co-expression of LAG-3 and PD-1 on tumor-infiltrating CD8(+) and CD4(+) T cells, and used anti-LAG-3/PD-1 dual antibodies to treat mice already resistant to single antibody treatment, and found that this combination cured most of the mice. Bottai et al. (118) found that PD-1 and LAG-3 were simultaneously expressed on tumor-infiltrating lymphocytes in approximately 15% of triple-negative breast cancer patients, providing an opportunity to evaluate anti-LAG and anti-PD-1/PD-L1. BMS-986016 (Relatlimab), the first IgG4 monoclonal antibody developed to target LAG-3, has been clinically tested in a variety of cancers and has been shown to be effective in combination with Nivolumab in advanced melanoma patients treated with anti-PD-1/anti-PD-L1 therapy. Sym022, MK-4280, REGN3767, TSR-033 and other LAG-3 inhibitors are in early clinical trials. Therapeutic combinations of different types of LAG-3 monoclonal antibodies in combination with PD-1/PD-L1 inhibitors will be used for cancer treatment in the future.

### Others (IDO, VISTA, TIGIT, B7/H3)

Indoleamine-2,3-dioxygenase (IDO) is an enzyme that converts tryptophan to kynurenine, which can affect cell survival. Thus, T cell proliferation is inhibited when tryptophan is lacking in the tumor microenvironment (119). Dill et al. (120) found that about 70% of TNBC L1 + breast cancer cells also expressed IDO, suggesting that IDO-related T cell damage may be the mechanism of anti-PD-1/PD-L1 immunotherapy drug resistance. Therefore, TNBC patients expressing IDO can be treated with PD-1/PD-L1 inhibitors combined with IDO inhibitors. A phase Ib clinical trial showed that 9% of patients treated with Atezolizumab combined with IDO inhibitor Navoximod reached partial response (PR) and 17% reached stable disease (SD). Most of the treatment-related adverse events were grade 1 and 2, of which the main ones were rash (22%) and fatigue (22%) (121). In addition to the these more commonly used immune checkpoint inhibitors mentioned above, TIGIT inhibitors, B7-H3 monoclonal antibodies, VISTA inhibitors, and other combinations with PD-1/PD-L1 inhibitors are also being evaluated in clinical trials.

## Combination With Chemotherapeutic Drugs

Traditionally, chemotherapeutic drug therapy relies on direct cytotoxic killing of tumor cells, and this non-selective killing often causes some damage to anti-tumor immune cells as well (122). As the research progresses, more and more studies show that some chemotherapeutic drugs, at certain doses, not only do not suppress the immune system, but also participate in the regulation of tumor immunosuppressive microenvironment, promote anti-tumor immune response, and enhance the anti-tumor effect with the immune system. Mathios et al. (123) found that in a mouse tumor model, although chemotherapy suppressed immune function, local chemotherapy can enhance the tumor immune response. This synergistic effect is particularly significant in the context of unsatisfactory immunotherapy alone. Chemotherapeutic drugs modulate the immune response mainly through the following mechanisms: (1) enhancing the immunogenicity of tumor cells. After killing tumor cells with chemotherapeutic drugs, tumor cells release a number of cell death-associated molecules (CDAMs), including high mobility group box 1 (HMGB1), adenosine triphosphate (ATP), calrectin (CRT), and so on. These CDAMs enter the tumor microenvironment or are exposed to dead tumor cells and can enhance their immunogenicity. (2) Enhancement sensitizes tumor cells to immune effector cells. Ramakrishnan et al. (124) found that the sensitivity of tumor cells to the killing effect of cytotoxic T lymphocytes was enhanced after the administration of some chemotherapeutic agents. This effect was associated with tumor cell surface death receptors [tumor necrosis factor-related apoptosis-inducing ligand recepor (TRAILR) and FAS (CD95)] (125) and mannose-6-phosphate receptors [mannose-6-phosphate receptors (MPR)] expression levels (126). In addition, Chen et al. (127) found that agranulocyte and doxorubicin could cause the disinhibition signal of T-cell activation, thus enhancing the killing effect of T cells on against tumor cells. (3) Enhancement of antigenicity of tumor cells. Jackaman et al. (128) found a significant increase in antigen recognition epitopes relative to pre-chemotherapy after the use of chemotherapy in mice inoculated with mesothelioma. The increase in antigenic epitopes was able to trigger cytotoxic T lymphocyte (CTL) killing of tumor cells. Among them, increased expression levels of MHC class I molecules are present on the surface of tumor cells after treatment with various chemotherapeutic agents, such as: cyclophosphamide, gemcitabine, oxaliplatin, paclitaxel, etc. (127). (4) Inhibition of Tregs and MDSCs and promotion of DCs function. Studies have shown that chemotherapeutic agents can remove the suppressive effect of regulatory T cells (Tregs) (129), reduce the number of myeloid derived suppressor cells (MDSCs) (127) and increase CD8+ T cell activity resulting in a more effective anti-tumor immune response. Meanwhile, Shurin and colleagues (130) found that low doses of various chemotherapeutic agents such as cyclophosphamide, vincristine, methotrexate, doxorubicin, paclitaxel, and doxorubicin enhanced the function of dendritic cells (DCs). Due to the failure of the immune system, the presence of chemotherapy-resistant tumor cells and the re-expansion of immunosuppressive cells, tumor patients are highly susceptible to recurrence after receiving chemotherapy. In this context, the addition of immunotherapy to chemotherapy regimens has the potential to serve to increase the sensitivity of tumors to immune checkpoint therapy and reduce the likelihood of tumor recurrence. Min et al. (131) found that other cytotoxic drugs such as 5-FU and paclitaxel could increase PD-L1 expression. This shows that the combination of the two can serve to increase the efficacy. In a 2017 study, 25 patients with advanced GC were treated with pembrolizumab combined with cisplatin and 5-fluorouracil (5-FU). The results showed that the ORR (PR+CR) of all patients was 60%. Compared with the known adverse reactions of chemotherapy, there was no significant difference in adverse reactions caused by combination therapy, and the clinical effect was good (132). Zhang et al. (133) conducted a study on the use of PD-1/PD-L1 inhibitors in separate inhibitors monotherapy and combined with nab-paclitaxel in patients with small cell carcinoma of the lung, found median OS values of 28.6 months and 15.9 months in the nab-paclitaxel and monotherapy groups, respectively. The results reflect the effectiveness of the combination, but this combination still needs to be further explored due to the lack of a control group using chemotherapy alone. A number of phase II/III clinical trials have been conducted in non-small cell lung to assess their efficacy and safety. In the III clinical trial NCT02578680, researchers found that pemetrexed plus pembrolizumab significantly improved PFS and OS in metastatic non-small cell lung cancer, and its tolerability and safety profile were also validated (134). Since the immunomodulatory effects of chemotherapeutic agents are related to the type of chemotherapeutic agent, the mode of administration, the dose, and the duration of immunotherapy received by patients, finding the optimal combination of chemotherapy with PD-1/PD-L1 inhibitors in patients with different tumors is an important future research direction (135). A phase III trial compared the efficacy of Atezolizumab combined with albumin-bound paclitaxel and albumin-bound paclitaxel plus placebo and found that PFS (7.5 months *vs* 5.0 months), OS (25 months *vs* 15.5 months) and ORR (53% v 33%) in the Atezolizumab group were higher than those in the control group among PD-L1 positive people. But this advantage is not obvious in PD-L1-negative people (136). Although this combination is theoretically well established, research on this area is still in the exploratory stage, and how to use the discovered mechanisms to design new dosing regimens to improve the antitumor effect of immunochemotherapy still needs to be investigated through a large number of trials. The effect of this combination on the incidence of adverse reactions is also of concern to researchers. West et al. (134) compared chemotherapy alone with Pembrolizumab in combination with chemotherapy for non-squamous non-small cell lung cancer and found that the incidence of grade 3 or higher adverse reactions was 66.8% and 71.9%, respectively, with grade 3 or higher adverse reactions being mainly anemia and neutropenia. Different dosing regimens also influenced the incidence of adverse reactions, and finding more efficient and less toxic combination regimens is also a direction for future research.

## Combination With Molecular Targeting Drugs

With the continuous development of tumor molecular biology, people have a deeper understanding of the biological characteristics of tumors and their molecular mechanisms. Some important driver molecules are involved in or regulate these biological properties of tumors, and small molecule targeted drugs developed for these driver molecules have made significant progress in the process of treating malignant tumors. PD-1/PD-L1 inhibitors rely on cytotoxic T cells for their antitumor effects, and the activation of cytotoxic T cells depends on tumor-specific antigens. Tumor-targeted drugs cause lysis of tumor cells, resulting in the exposure of a large number of tumor-specific antigens that activate T cells to become effector T cells. Molecularly targeted drugs not only restore T cell activity and mediate tumor antigen release, but also regulate PD-L1 expression on the surface of tumor cells (137). The combination of these two drugs has the potential to increase the efficacy, so there are high expectations for the efficacy of combination therapy with molecularly targeted drugs (138).

### Vascular Endothelial Growth Factor (VEGF) Inhibitors

Tumor vasculature is highly abnormal compared to normal tissue vasculature, and the main cause of vascular abnormalities is the overexpression of VEGF. Immunosuppression and angiogenesis are two processes that are closely related during tumor development. Vasculature in tumors can exert immunosuppressive effects through various mechanisms, the four most important of which are: (1) promoting the recruitment of immunosuppressive cells (tumor-associated macrophages, myeloid-derived suppressor cells, regulatory cells) and proliferation (139, 140). (2) Inhibit the proliferation, transport and function of cytotoxic T lymphocytes (141). (3) Promoting abnormal tumor angiogenesis, leading to low pH and hypoxia in the tumor microenvironment, which subsequently causes immunosuppression systemically and locally (142, 143). (4) Inhibit antigen presentation and dendritic cell maturation, thereby hindering T cell activation (144, 145). PD-1/PD-L1 inhibitors can normalize tumor vasculature by promoting γ-interferon production and activating effector T cells, thereby enhancing the killing function of effector T cells and the efficacy of VEGF inhibitors. The combination of PD-1/PD-L1 inhibitors and VEGF inhibitors has been extensively studied in patients with various cancers. In 2019, the combination of pembrolizumab and axitinib was approved as a first-line agent in advanced renal cell carcinoma (146). Uemura et al. (147) evaluated two regimens for the treatment of advanced renal cell carcinoma, (avelumab + axitinib) and sunitinib, and found that patients treated with avelumab + axitinib had higher objective remission rates (ORR) and progression-free survival (PFS). However, it has also been shown that high doses of VEFG inhibitors in combination with PD-1/PD-L1 inhibitors can directly disrupt tumor vasculature, which can lead to more severe hypoxia and thus exacerbate tumor progression (148). A study of untreated patients with advanced NSCLC treated with an anti-VEGFR monoclonal antibody in combination with Pembrolizumab showed an incidence of grade 3+ treatment-related adverse events of 42.3%, with hypertension (15.4%) and acute myocardial infarction (7.7%) being the two most common adverse reactions (149). Results from another study comparing the Atezolizumab group with the Atezolizumab combined with Bevacizumab group showed that the incidence of grade 3+ TRAEs was 5% and 20% in the two groups, respectively. Of these, the most common adverse reactions in the combination group were hypertension (5%) and proteinuria (3%) (150). Currently, clinical data on the combination of PD-1/PD-L1 inhibitors with VEGF inhibitors for the treatment of cancer are scarce, and future studies will focus mainly on determining the optimal combination and dose of VEGF inhibitors and PD-1/PD-L1 inhibitors for better anti-cancer efficacy.

### Epidermal Growth Factor Receptor (EGFR) Inhibitors

EGFR belongs to the tyrosine kinase receptor (ErbB) family, a cell surface receptor encoded by the HER1 (ErbB1) gene. binding of the extracellular portion of EGFR and extracellular ligands allows EGFR monomers to form dimers with other EGFR molecules or other ErbB family protein receptors. Dimer phosphorylation promotes tumor cell proliferation, differentiation and migration through activation of the downstream signaling pathways RAS-PI3K-PTEN-AKT-mTOR and RAS-RAF-MEK-ERK (151). Mutations and excess phenotypes or mutations of EGFR are associated with aggressive tumor behavior. including treatment resistance and metastasis of tumor cells. Its high expression is particularly evident in breast cancer (152, 153). However, researchers have found that its effectiveness in treating cancer as a single agent does not meet expectations. Akbay et al. (76) found that mutations in EGFR in NSCLC cause high expression of PD-L1, allowing tumor cells to evade the immune system through the PD-1/PD-L1 pathway. Consistent with these findings, Li et al. (55) found that EFGR inhibitors such as lapatinib, erlotinib and gefitinib can reduce epidermal growth factor-induced PD-L1 expression and thus achieve anti-tumor effects. These findings also illustrate the feasibility of combining EGFR inhibitors and PD-1/PD-L1 inhibitors for cancer treatment. Sugiyama et al. (154) used an anti-PD-1 monoclonal antibody in combination with an EGFR inhibitor in a mouse model of lung cancer and found its efficacy to be better than either treatment alone. However, in a retrospective study, researchers found that 15% of patients who used PD-L1 antibodies followed by the combination of oxitinib experienced immune-related adverse effects. And the shorter the interval between the use of both, the higher the probability of adverse reactions (155). A phase I trial evaluating Atezolizumab in combination with Erlotinib in patients with NSCLC showed a 39% incidence of grade 3 or higher treatment-related adverse events among patients, with fever, rash, and elevated alanine transaminases each accounting for 7% of the most common (156). Therefore, the combination of PD-1/PD-L1 inhibitors with egfr inhibitors needs to be used with caution while avoiding the occurrence of adverse reactions.

### Poly ADP-Ribose Polymerase (PARP) Inhibitors

PARP is a poly ADP ribose polymerase present in eukaryotic cells, which is involved in the repair of dna damage and the regulation of apoptosis (157). Also, PARP is involved in the regulation of cellular signal transduction, transcriptional and methylation modification of dna, and cell cycle regulation. DNA damage activates a large amount of PARP to initiate repair function, and PARP inhibitors are effective in preventing DNA repair in cancer cells by inhibiting the function of PARP (158). However, most patients are resistant to PARP inhibitors, resulting in no significant improvement in the overall survival of patients treated with monotherapy (159). PAPR inhibitors promote the expression of PD-L1 to regulate the tumor immune microenvironment, leading to tumor cell resistance to the killing effect of toxic t cells. This also suggests that PD-1/PD-L1 inhibitors are capable of synergistic effects with them (160). The current study found that the use of a combination of PD-1/PD-L1 inhibitors and parp inhibitors in patients with triple-negative breast cancer and ovarian cancer inhibited tumor cell proliferation (161, 162). In a recent study, Wu et al. (163) conducted a retrospective analysis of 40 patients with solid tumors using a combination of PARP inhibitors and PD-L1 inhibitors. They analyzed the efficacy metrics [including overall survival (OS), disease control rate (DCR), progression-free survival (PFS) and objective remission rate (ORR)] and safety metrics (occurrence of grade 3 or greater adverse events) of nivolumab/pembrolizumab in combination with niraparib/olaparib and found that this combination efficacy metrics were better than with PD-1 inhibitors alone. In addition, the combination was found higher ORR in patients with gynecologic tumors and small cell lung cancer (164, 165). In 2018, the FDA approved Olaparib for use in metastatic breast cancer with mutations in the breast cancer susceptibility gene (BRCA) and human epidermal growth factor receptor 2 (HER2) negativity. By testing PD-1/PD-L1 inhibitors in combination with PARP inhibitors in a variety of cancers, the use of PARP inhibitors will become more widespread in the future.

### CDK4/6 Inhibitors

Cancer is also considered to be a cell cycle disease (166) as cell cycle dysregulation is present in all human cancers and tumor cells often show genetic instability and abnormal proliferation. To date, scientists have conducted a series of studies on cell cycle regulation mechanisms, which have led to the emergence of anticancer drugs targeting the cell cycle as a hot topic of research. The CDK4/6-RB-P16 signaling pathway plays an important role in the regulation of the cell cycle. Abnormal proliferation of tumor cells resulting from abnormal regulation of this pathway is seen in various cancers (167). FDA has approved CDK4/6 inhibitors in HER2-negative, ER-positive postmenopausal breast cancer patients, which can block the binding site of cell cycle protein D1 on CDK4/6 thereby inhibiting tumor cell proliferation. Teh et al. (168) found that CDK4/6 inhibitors also could achieve anticancer effects by increasing the immunogenicity of tumors and downregulating immunosuppressive cells. However, CDK4/6 inhibitors alone are prone to drug resistance, and in order to be able to expand their use, researchers have conducted trials of various combination therapies. Goel et al. (169) found that the use of CDK4/6 inhibitors in patients with breast and colorectal cancer enhanced the sensitivity of tumor cells to anti-PD-1/PD-L1 antibodies through preclinical studies. However, further studies are needed to confirm the effectiveness of CDK4/6 inhibitors in combination with PD-1/PD-L1 inhibitors due to the lack of convincing trial data. To investigate whether CDK4/6 inhibitors and PD-1/PD-L1 inhibitors can act synergistically, Zhang et al. (56) constructed ovarian cancer mouse models and treated them with abemaciclib monotherapy and combined anti-PD-1 therapy, respectively. The results showed that the combination therapy provided better control of tumor cells relative to the single-agent group. In recent years, researchers have also found that CDK4/6 inhibitors improve the tumor immune microenvironment and enhance T-cell activity, and have been found to significantly increase the antitumor effect when combined with PD-1/PD-L1 inhibitors in a variety of animal models of cancer (168, 170).These studies also illustrate the potential of combining the two in the future treatment of cancer.

### Other Molecular Targeted Drugs

In addition to some of the drugs described above, the combination of molecularly targeted drugs such as BRAF inhibitors, MEK inhibitors, and adenosine receptor inhibitors with PD-1/PD-L1 inhibition has joined the research process of researchers. Mutations in the BRAF gene are closely associated with the development of melanoma, yet most melanoma patients are resistant to BRAF inhibitors (171). Frederick et al. (172) found this phenomenon to be associated with upregulation of PD-L1 expression. This also suggests the possibility that PD-1/PD-L1 inhibitors can increase the efficacy of BRAF inhibitors. Nevertheless, satisfactory results were not obtained in some clinical trials in melanoma patients using this combination (173, 174). When BRAF is mutated it activates the downstream MAPK pathway, leading to the activation of MEK, ERK and other proteins, which in turn regulate the processes of cell proliferation, growth and apoptosis (175). Researchers have found in clinical studies that the use of BRAF inhibitors in combination with MEK inhibitors in patients with metastatic melanoma can reduce the development of resistance to BRAF inhibitors in patients, thereby enhancing the therapeutic effect (176). Several investigators have used preclinical models to find that combining anti-PD-1 therapy with BRAF inhibitors and MEK inhibitors improves efficacy (177, 178). In a subsequent clinical trial, Ribas et al. (179) evaluated the safety and efficacy of this triple combination therapy and showed that it had a longer antitumor effect and PFS. although the probability of adverse events was also increased, this combination may be best suited for patients who do not respond well to monotherapy. The sequence and optimal duration of therapy for the combination of these three agents remains unclear, and their long-term efficacy requires continued attention, so longer follow-up and more studies are needed to assess the safety and efficacy of this combination. Adenosine is an immunomodulatory molecule that, when combined with adenosine 2A receptor (A2AR) and adenosine 2B receptor (A2BR), enhances the function of immunosuppressive cells (e.g., MDSCs and Tregs) and inhibits the activation of immunoprotective cells (e.g., dendritic cells, T cells, and NK cells), resulting in an immunosuppressive effect (180). Based on the ability of adenosine to inhibit T-cell function and the fact that upregulation of adenosine receptor expression levels can be found on activated T-lymphocytes after blockade of PD-1 has been shown (181), this also suggests that PD-1/PD-L1 inhibitors combined with adenosine receptor inhibitors have the potential to control tumorigenesis and progression. After Beavis et al. (182) evaluated this combination therapy in a mouse model of cancer, they found that this combination promoted IFNγ production by tumor-infiltrating CD8+ T lymphocytes. also they found that the tumor size in the combination group was significantly smaller than in the PD-1 monotherapy group, and that this potentiation was associated with IFNγ production. It is important to note that different dosing sequences of ICIs and small-molecule-targeted drugs may result in widely varying efficacy and adverse effects.

## Other Combinations

Immunomodulators can activate one or more immune active cells and increase the body’s specific and non-specific immune function. It can restore conditions caused by immune deficiency and is often used as adjuvant therapy for immunodeficiency diseases or malignancies. Promotion of IL-2 production was the first immune regimen approved for cancer therapy and currently mediates immunomodulation by activating IL-2 receptor trimers (CD25, CD132 and CD122) and dimers (CD132 and CD122) (183). IL-2 has been approved for the treatment of metastatic melanoma and renal cancer since its use in cancer therapy in 1984 to date patients. However, the short half-life of IL-2 requires the use of larger doses, which increases the probability of adverse effects. With the advent of various immunotherapies, researchers have also begun to explore different combinations of IL-2 for treatment. Monotherapy with NKTR-214 (an agonist targeting CD122) in cancer patients revealed increased expression of PD-1 and PD-L1 on the cell surface, along with a significant increase in NK cells and CD8+ T cells in patients, suggesting a potential synergistic mechanism of action of PD-1 inhibitors with NKTR-214 (184). Diab et al. (185) in solid tumors (kidney cancer, melanoma, uroepithelial cancer, triple-negative breast cancer and non-small cell lung cancer) in a phase I/II clinical trial of Nivolumab in combination with NKTR-214, which found ORRs of 50%, 54% and 52% for the combination in patients with non-small cell lung cancer, kidney cancer and melanoma, respectively. Selective class I histone deacetylase inhibitors (HDACIs), a class of compounds with the ability to interfere with the function of histone deacetylases, have also been shown to have anti-tumor angiogenic effects and inhibitory effects on tumor cell metastasis, migration and invasion. It increases the immunogenicity of tumor cells by activating the expression of costimulatory molecules and antigenic presentation of tumor antigens in tumor cells (186, 187). Orillion et al. (188) found that entesta could enhance the anti-PD-1 antitumor effect by getting rid of the immunosuppressive tumor microenvironment and providing functional inhibition of myeloid-derived suppressor cells (MDSCs). Clinical trials are currently evaluating the efficacy of PD-L1 inhibitors in combination with HDACi in patients with advanced TNBC (NCT02708680). In addition, researchers have identified a number of cytokines that promote tumor growth, such as IL-6 and IL-17A. Some preclinical trials have demonstrated a synergistic effect of the relative inhibitors in combination with PD-1/PD-L1 inhibitors (189, 190).

The discovery of several co-stimulatory targets in the tumor necrosis factor receptor (TNFR) superfamily has also facilitated the development of corresponding antitumor drugs (191). These drugs can act synergistically with immune checkpoint drugs to jointly enhance the immune response induced by checkpoint inhibition. These costimulatory receptors are expressed on CD8+ T cells and CD4+ T cells and include CD137, OX40, GITR and CD27. CD137, a member of the TNF receptor superfamily, promotes the activation of NK cells and T cells when its expression is upregulated and can act synergistically when combined with anti-PD-1 therapy in tumor patients (192). Chen et al. (193) evaluated the combination of anti-PD-1 with anti-LAG3 and anti-CD137 with PD-1 in a mouse model of B16F10 melanoma and showed that simultaneous blockade of PD-1 and CD137 had the most pronounced tumor suppressive effect. Also the synergistic antitumor effect of both combinations was demonstrated in MC38 colon cancer model mice. Although the efficacy of the combination of PD-L1/PD-1 and CD137 in the clinical setting is currently unknown, further clinical trials are warranted based on the effects assessed in tumor mouse models. The combination of CD27 antibody (varlilumab) with Nivolumab has also passed a phase I trial, showing that the enrolled 3 cases of head and neck squamous carcinoma, 4 cases of melanoma, 8 cases of ovarian cancer and 21 colorectal cancer patients showed 11 cases of stable disease (SD) and 3 cases of partial remission (PR) (194). Early clinical trials evaluating the safety of PD-1 antibodies in combination with CD137 and CD27 are currently underway, but no data have yet been published. Researchers have found that OX40 can be expressed in tumor-infiltrating lymphocytes in breast cancer, and several studies have shown that breast cancer development and progression are closely associated with OX40L (195). Many OX40 agonists have been used in combination with PD-1/PD-L1 inhibitors in clinical trials for hematologic malignancies and various solid tumors, which are still recruiting or awaiting results (NCT04198766, NCT03323398, NCT03390296, NCT03894618, NCT03217747 NCT04730843).

Chimeric antigen receptor (CAR) is a new type of receptor formed by integrating multiple domains of different proteins by means of genetic engineering. It can bind to specific antigens and induce specific effector molecules to perform biological functions. After the gene encoding CAR was transfected into T lymphocytes for gene modification, the result was Chimeric-antigen receptor T cell (CAR-T). It can kill tumor cells specifically by recognizing specific antigens on the surface of tumor cells, so as to achieve the purpose of tumor elimination (196). CAR-T therapies are currently playing an outstanding role in the treatment of hematologic malignancies. There have been CAR-T cell therapeutics approved by the FDA for the treatment of diffuse large B-cell lymphoma and acute lymphoblastic leukemia (196, 197). The anti-tumor efficacy of CAR-T cells is closely related to their number and function. It has been found that inhibition of PD-1 receptor can attenuate the inhibition of CAR-T cells by PD-1 pathway and enhance the proliferation of CAR-T cells (198). Moreover, the anti-tumor activity of CAR-T cells was enhanced when the PD-1 gene was knocked out (199). After designing CAR-T constitutive cells that secrete anti-PD-1 antibodies, some researchers found that the blocking effect of PD-1 antibodies attenuated suppressive T-cell signaling and enhanced T-cell proliferation and effector functions both *in vitro* and *in vivo*. This also shows that the combination of the two has better efficacy. Elise et al. (200) added PD-1 monoclonal antibody to 12 patients with diffuse large B-cell lymphoma who had progressed after CAR-T treatment and found that 75% of patients showed expansion of CAR-T cells, in addition to two cases achieving partial response and one case achieving complete response. More clinical trials are needed in the future to expand this combination therapy to more tumors.

Epigenetics is a phenomenon that occurs widely in tumors and plays an important role in the formation and maintenance of malignancy heterogeneity (201). In addition, a growing number of studies show that tumors can evade the immune system through epigenetic modulation (202). Epigenetic inhibitors are emerging anti-cancer drugs that have emerged in recent years. There are three major classes of epigenetic inhibitors in common use: histone deacetylase inhibitors (HDACi), DNA methyltransferase inhibitors (DNMTi) and histone methyltransferase inhibitors (HMTi). They have been shown to upregulate PD-L1 expression in tumor tissue while inhibiting the activity of those cells surrounding the tumor that suppress the anti-cancer immune response, such as MDSC cells (83). However, the use of epigenetic inhibitors alone can only weakly stimulate the immune system, and tumors can still inhibit the immune response, so the therapeutic effect is limited (203). The combination of epigenetic inhibitor and PD-1/PD-L1 inhibitor can effectively slow down tumor progression and improve survival rate. Nie et al. (204) evaluated the efficacy of PD-1 monotherapy and PD-1 combined with DNA methyltransferase inhibitor (decitabine) in patients with relapsed/refractory classical Hodgkin’s lymphoma. The results showed that the complete remission rate of the combined group was higher than that of the monotherapy group (71% *vs* 32%). In addition, the efficacy retention rate of the combined group was also higher than that of the monotherapy group (100% *vs* 76%). These data also show that decitabine may have the ability to sensitize PD-1 and overcome drug resistance. At present, the combination of epigenetic drugs and PD-1 monoclonal antibody is still in the early clinical exploration stage, and a large number of clinical trials are recruiting patients (NCT03590054, NCT03812796, NCT03337698, NCT03179943, NCT03390296, NCT03854474, NCT03161223, NCT03346642, etc.).

## Conclusion and Perspectives

**Figure 3:**
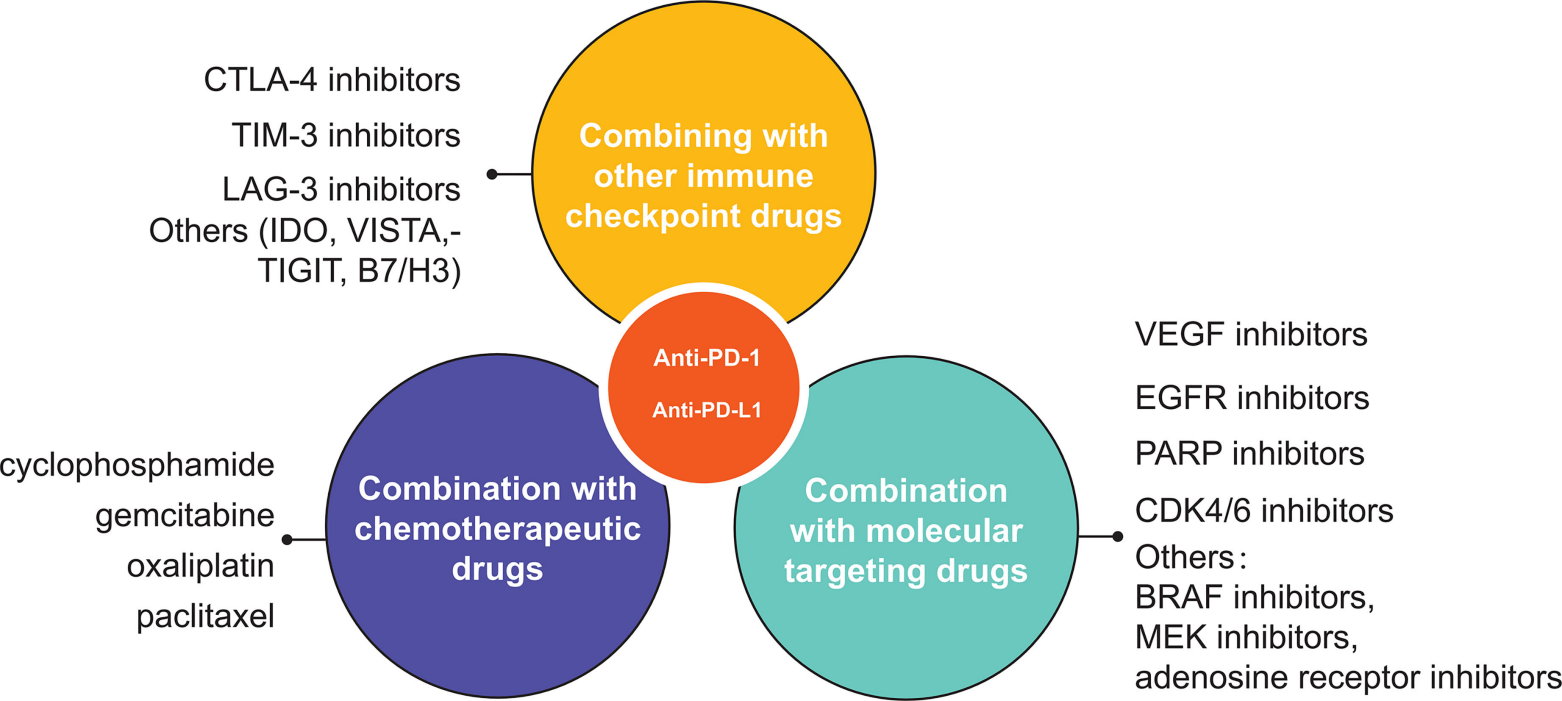
A schematic overview of various uses of PD1/PD-L1 inhibitor in oncology. The three main types of combination are other immune checkpoint inhibitors, chemotherapy and molecular targeted drugs.

With the emergence and innovation of new technologies and the increasing cross-fertilization between different disciplines, more and more drugs are being used alone and in combination in cancer therapy (**Figure 3**). The search for appropriate combinations to improve prognosis and increase tumor response rates is a hot topic of research today. A large number of studies have shown that PD-1/PD-L1 combination therapy needs to be tailored to the characteristics of each cancer, draw on and incorporate existing research findings, screen for efficacious single agents and therapies, further develop synergistic combination strategies and implement them gradually in clinical trials. It is not possible to simply copy and paste other trial designs. Compared to well-established single-agent clinical trials, combination therapies often lack sufficient clinical trial data on safety and efficacy. Also, the unique mechanism of action of PD-1/PD-L1 inhibitors may increase the risk of combination drug use. Therefore, the uncertainty and complexity of combination therapy need to be highly valued before conducting clinical trials, and the rationality of combination therapy needs to be fully demonstrated before conducting trials. To date a number of PD-1/PD-L1 inhibitor-based combinations of various combinations have completed Phase II/III clinical trials (**Table 3**). The preliminary clinical data obtained are impressive, but many issues still need to be addressed, including adverse effect prediction, indications, applicable population, dosing schedule, dosing dose, combination sequence, and efficacy evaluation criteria. The emergence of nanomedicine has provided a new direction for cancer immunotherapy. Combining drugs with nanoparticles enables the precise targeting of drugs to tumors, thus amplifying the therapeutic effect of drugs. In addition, the emergence of bifunctional antibodies with better targeting and effective synergistic effects through dual pathway or dual target blockade has given new insights into cancer therapy, which may become one of the key therapeutic strategies for mankind to overcome cancer.

**Table 3:** Various uses of PD1/PD-L1 inhibitor in oncology.

Immune Checkpoint Inhibitor	Combination Therapy	Tumour Type	Phase	Primary Outcome			NCT Number
				Title	Experimental group	Control group	
**Chemotherapy**							
Atezolizumab	Carboplatin+Etoposide	Extensive-Stage (ES) Small Cell Lung Cancer	3	PFS	5.2 (4.4 to 5.6)	4.3 (4.2 to 4.5)	NCT02763579
				OS	12.3 (10.8 to 15.9)	10.3 (9.3 to 11.3)
Atezolizumab	Nab-Paclitaxel+ Carboplatin	Stage IV Non-Squamous Non-Small Cell Lung Cancer	3	PFS	7.0 (6.2 to 7.3)	5.5 (4.4 to 5.9)	NCT02367781
				OS	18.6 (16.0 to 21.2)	13.9 (12.0 to 18.7)
Atezolizumab	Bevacizumab + Paclitaxel + Carboplatin	Stage IV Non-Squamous Non-Small Cell Lung Cancer	3	PFS	8.3 (7.7 to 9.8)	6.8 (6.0 to 7.1)	NCT02366143
				OS	19.2 (17.0 to 23.8)	14.7 (13.3 to 16.9)
Atezolizumab	Paclitaxel	Advanced or Metastatic Triple Negative Breast Cancer	3	PFS	5.95 (5.62 to 7.43)	5.72 (5.39 to 7.20)	NCT03125902
Atezolizumab	Nab-Paclitaxel + Carboplatin	Advanced Squamous Non-Small Cell Lung Cancer	3	PFS	6.5 (5.7 to 7.1)	5.6 (5.5 to 5.7)	NCT02367794
				OS	14.2 (12.3 to 16.8)	13.5 (12.2 to 15.1)
Atezolizumab	Carboplatin or Cisplatin + Pemetrexed	Stage IV Non-Squamous Non-Small Cell Lung Cancer	3	PFS	7.7 (6.7 to 8.5)	5.2 (4.3 to 5.6)	NCT02657434
Durvalumab	Carboplatin or Cisplatin+ Etoposide	Untreated Extensive-Stage Small Cell Lung Cancer	3	OS	13.0 (11.5 to 14.8)	10.3 (9.3 to 11.2)	NCT03043872
Durvalumab	Nab-Paclitaxel	Advanced Non-small Cell Lung Cancer	2	PFS	4.5 (3.45 to 5.88)	4.2 (2.79 to 5.06)	NCT02250326
Pembrolizumab	pemetrexed	Metastatic Non-Small-Cell Lung Cancer	3	PFS	8.8 (7.6 to 9.2)	4.9 (4.7 to 5.5)	NCT02578680
Pembrolizumab	Etoposide	Extensive Stage Small Cell Lung Cancer	3	PFS	4.8 (4.3 to 5.4)	4.3 (4.2 to 4.5)	NCT03066778
				OS	10.8 (9.2 to 12.9)	9.7 (8.6 to 10.7)
Pembrolizumab	Carboplatin-Paclitaxel/Nab-Paclitaxel	Metastatic Squamous Non-small Cell Lung Cancer	3	PFS	6.4 (6.2 to 8.3)	4.8 (4.2 to 5.7)	NCT02775435
Pembrolizumab	Pemetrexed+Platinum	Metastatic Nonsquamous Non-small Cell Lung Cancer	3	PFS	8.8 (7.6 to 9.2)	4.9 (4.7 to 5.5)	NCT02578680
Pembrolizumab	Cisplatin+ 5-FU/ Capecitabine	Advanced Gastric or Gastroesophageal Junction Adenocarcinoma	3	PFS	6.9 (5.7 to 7.3)	6.4 (5.7 to 7.0)	NCT02494583
				OS	12.5 (10.8 to 13.9)	11.1 (9.2 to 12.8)
Pembrolizumab	Docetaxel	Non-Small Cell Lung Cancer	2	ORR	42.5 (26.9 to 58.1)	15.8 (4.0 to 27.6)	NCT02574598
Pembrolizumab	Carboplatin+ Pemetrexed	Non-small Cell Lung Cancer	2	ORR	55.0 (41.6 to 67.9)	28.6 (17.9 to 41.3)	NCT02039674
Pembrolizumab	Docetaxel	Non-Small Cell Lung Cancer	2	ORR	42.5 (26.9 to 58.1)	15.8 (4.0 to 27.6)	NCT02574598
Pembrolizumab	Carboplatin+ Pemetrexed	Non-small Cell Lung Cancer	2	ORR	55.0 (41.6 to 67.9)	28.6 (17.9 to 41.3)	NCT02039674
**Targeted therapy**							
Atezolizumab	Bevacizumab	Advanced Renal Cell Carcinoma	3	PFS	7.5 (6.8 to 9.7)	11.2 (8.6 to 14.3)	NCT02420821
Atezolizumab	Cobimetinib	Metastatic Colorectal Adenocarcinoma	3	OS	8.87 (7.00 to 10.61)	8.51 (6.41 to 10.71) 7.10 (6.05 to 10.05)	NCT02788279
Atezolizumab	Tiragolumab	Locally Advanced or Metastatic Non-Small Cell Lung Cancer	2	ORR	31.3 (19.49 to 43.20)	16.2 (6.69 to 25.66)	NCT03563716
Atezolizumab	Bevacizumab	Untreated Advanced Renal Cell Carcinoma	2	PFS	11.7 (8.4 to 17.3)	6.1 (5.4 to 13.6)	NCT01984242
Atezolizumab	Bevacizumab+Capecitabine	Refractory Metastatic Colorectal Cancer	2	PFS	4.37 (4.07 to 6.41)	3.32 (2.14 to 6.21)	NCT02873195
Durvalumab	Tremelimumab	Advanced Colorectal Cancer	2	OS	6.6 (6.0 to 7.4)	4.1 (3.3 to 6.0)	NCT02870920
Pembrolizumab	Epacadostat	Recurrent or Metastatic Head and Neck Squamous Cell Carcinoma	3	ORR	31.4 (16.9 to 49.3)	21.1 (6.1 to 45.6)	NCT03358472
Pembrolizumab	Epacadostat	Cisplatin-ineligible Urothelial Carcinoma	3	ORR	31.8 (22.46 to 55.24)	24.5 (15.33 to 43.67)	NCT03361865
Pembrolizumab	Axitinib	Renal Cell Carcinoma	3	PFS	15.1 (12.6 to 17.7)	11.0 (8.7 to 12.5)	NCT02853331
Nivolumab	Ipilimumab	Untreated Advanced Melanoma	3	PFS	11.50 (8.90 to 16.72)	6.87 (4.34 to 9.46) 2.89 (2.79 to 3.42)	NCT01844505
Nivolumab	Ipilimumab	Advanced or Metastatic Renal Cell Carcinoma	3	ORR	41.6 (36.9 to 46.5)	26.5 (22.4 to 31.0)	NCT02231749
Nivolumab	Ipilimumab	Non-Small Cell Lung Cancer	3	OS	14.13 (13.24 to 16.16)	10.74 (9.46 to 12.45)	NCT03215706
Nivolumab	Ipilimumab	Untreated Advanced or Metastatic Renal Cell Carcinoma	3	ORR	41.6 (36.9 to 46.5)	26.5 (22.4 to 31.0)	NCT02231749
				PFS	11.56 (8.71 to 15.51)	8.38 (7.03 to 10.81)
Pembrolizumab	Acalabrutinib	Advanced Non-small Cell Lung Cancer	2	OR	4 ( 14.3%)	4 ( 12.9%)	NCT02448303
Pembrolizumab	Acalabrutinib	Ovarian Cancer	2	OR	3 ( 9.1% )	1 ( 2.9% )	NCT02537444
Pembrolizumab	Acalabrutinib	Advanced or Metastatic Pancreatic Cancer	2	OR	3 ( 11.1%)	0 ( 0.0%)	NCT02362048
Nivolumab OR Pembrolizumab	Glembatumumab	Advanced Melanoma	2	ORR	14.3	11.3	NCT02302339
Nivolumab	Ipilimumab	Untreated, Unresectable, or Metastatic Melanoma	2	ORR	59.7 (47.5 to 71.1)	10.8 (3.0 to 25.4)	NCT01927419
Nivolumab	Ipilimumab	Persistent or Recurrent Epithelial Ovarian, Primary Peritoneal, or Fallopian Tube Cancer	2	ORR	31.4 (21 to 44)	12.2 (5 to 23)	NCT02498600
Nivolumab	Andecaliximab	Unresectable or Recurrent Gastric or Gastroesophageal Junction Adenocarcinoma	2	ORR	9.7 (4.0 to 19.0)	6.9 (2.3 to 15.5)	NCT02864381
Pembrolizumab	Acalabrutinib	Advanced Non-small Cell Lung Cancer	2	OR	4 ( 14.3%)	4 ( 12.9%)	NCT02448303
Pembrolizumab	Acalabrutinib	Ovarian Cancer	2	OR	3 ( 9.1% )	1 ( 2.9% )	NCT02537444
Pembrolizumab	Acalabrutinib	Advanced or Metastatic Pancreatic Cancer	2	OR	3 ( 11.1%)	0 ( 0.0%)	NCT02362048
Nivolumab OR Pembrolizumab	Glembatumumab	Advanced Melanoma	2	ORR	14.3	11.3	NCT02302339
Nivolumab	Ipilimumab	Untreated, Unresectable, or Metastatic Melanoma	2	ORR	59.7 (47.5 to 71.1)	10.8 (3.0 to 25.4)	NCT01927419
Nivolumab	Ipilimumab	Persistent or Recurrent Epithelial Ovarian, Primary Peritoneal, or Fallopian Tube Cancer	2	ORR	31.4 (21 to 44)	12.2 (5 to 23)	NCT02498600
Nivolumab	Andecaliximab	Unresectable or Recurrent Gastric or Gastroesophageal Junction Adenocarcinoma	2	ORR	9.7 (4.0 to 19.0)	6.9 (2.3 to 15.5)	NCT02864381
**Others**							
Avelumab	Pegylated Liposomal Doxorubicin (PLD)	Platinum Resistant/Refractory Ovarian Cancer	3	OS	15.7 (12.7 to 18.7)	11.8 (8.9 to 14.1) 13.1 (11.8 to 15.5)	NCT02580058
				PFS	3.7 (3.3 to 5.1)	1.9 (1.8 to 1.9) 3.5 (2.1 to 4.0)
Atezolizumab	Trastuzumab Emtansine	Human Epidermal Growth Factor-2 (HER2) Positive Locally Advanced or Metastatic Breast Cancer (BC) Who Received Prior Trastuzumab and Taxane Based Therapy (KATE2)	2	PFS	8.2 (5.8 to 10.7)	6.8 (4.0 to 11.1)	NCT02924883
Nivolumab	Pegilodecakin	Metastatic Non-Small Cell Lung Cancer	2	ORR	14.8 (4.2 to 33.7)	12.0 (2.6 to 31.2)	NCT03382912
Nivolumab	Pegilodecakin	Metastatic Non-Small Cell Lung Cancer	2	ORR	14.8 (4.2 to 33.7)	12.0 (2.6 to 31.2)	NCT03382912

## Author Contributions

There are 3 first authors in this manuscript and they have equally contributed to this project. ZL, GQS, and GSS were responsible for gathering information of the related research and designing the review. YC, LW, and QW were responsible for drawing the pictures. Furthermore, we have four corresponding authors in this manuscript. WT and YX have contributed to information interpretation, editing and critical revision of the manuscript. CL, WT, and YZ have contributed to study design and critical revision of the manuscript. All authors contributed to the article and approved the submitted version.

## Funding

This work was supported by grants from National Natural Science Foundation of China (Grant No.81771716). We also greatly thank the China Scholarship Council (CSC) for supporting QW (No. 202008320313).

## Conflict of Interest

The authors declare that the research was conducted in the absence of any commercial or financial relationships that could be construed as a potential conflict of interest.

## Publisher’s Note

All claims expressed in this article are solely those of the authors and do not necessarily represent those of their affiliated organizations, or those of the publisher, the editors and the reviewers. Any product that may be evaluated in this article, or claim that may be made by its manufacturer, is not guaranteed or endorsed by the publisher.
